# Inflammation and adipose tissue: effects of progressive load training in rats

**DOI:** 10.1186/1476-511X-9-109

**Published:** 2010-10-04

**Authors:** Fábio S Lira, José C Rosa, Gustavo D Pimentel, Victor AF Tarini, Ricardo M Arida, Flávio Faloppa, Eduardo S Alves, Cláudia O do Nascimento, Lila M Oyama, Marília Seelaender, Marco T de Mello, Ronaldo VT Santos

**Affiliations:** 1 Department of Physiology of Nutrition, Universidade Federal de São Paulo, São Paulo, Brazil; 2 Department of Orthopaedics and Traumatology, Universidade Federal de São Paulo, São Paulo, Brazil; 3 Department of Physiology, Universidade Federal de São Paulo (UNIFESP), São Paulo, SP, Brazil; 4 Department of Bioscience, Universidade Federal de São Paulo, Baixada Santista Campus, São Paulo, Brazil; 5 Department of Psychobiology, Universidade Federal de São Paulo - UNIFESP, São Paulo, Brazil; 6 Cancer Metabolism Research Group, Institute of Biomedical Sciences, University of São Paulo, São Paulo, Brazil

## Abstract

**Introduction:**

Cytokines (IL-6, IL-10 and TNF-α) are increased after exhaustive exercise in the rat retroperitoneal (RPAT) and mesenteric adipose tissue (MEAT) pads. On the other hand, these cytokines show decreased expression in these depots in response to a chronic exercise protocol. However, the effect of exercise with overload combined with a short recovery period on pro- and anti-inflammatory cytokine expression is unknown. In the present study, we investigated the regulation of cytokine production in the adipose tissue of rats after an overtraining-inducing exercise protocol.

**Methods:**

Male Wistar rats were divided into four groups: Control (C), Trained (Tr), Overtrained (OT) and recovered overtrained (R). Cytokines (IL-6, TNF-α and IL-10) levels and Toll Like Receptor 4 (TLR4), Nuclear Factor kBp65 (NF-kBp65), Hormone Sensitive Lipase (HSL) and, Perilipin protein expression were assessed in the adipose tissue. Furthermore, we analysed plasma lipid profile, insulin, testosterone, corticosterone and endotoxin levels, and liver triacylglycerol, cytokine content, as well as apolipoprotein B (apoB) and TLR4 expression in the liver.

**Results:**

OT and R groups exhibited reduced performance accompanied by lower testosterone and increased corticosterone and endotoxin levels when compared with the control and trained groups. IL-6 and IL-10 protein levels were increased in the adipose tissue of the group allowed to recover, in comparison with all the other studied groups. TLR-4 and NF-kBp65 were increased in this same group when compared with both control and trained groups. The protein expression of HSL was increased and that of Perilipin, decreased in the adipose in R in relation to the control. In addition, we found increased liver and serum TAG, along with reduced apoB protein expression and IL-6 and IL-10 levels in the of R in relation to the control and trained groups.

**Conclusion:**

In conclusion, we have shown that increases in pro-inflammatory cytokines in the adipose tissue after an overtraining protocol may be mediated via TLR-4 and NF-kBp65 signalling, leading to an inflammatory state in this tissue.

## Introduction

The current view of the function of white adipose tissue (WAT) envisages its secretory properties in addition to lipid storage [1]. WAT actively secretes various bioactive peptides, termed "adipokines", which act locally and distally with autocrine, paracrine and endocrine effects [2].

TNF-α is the most-studied cytokine in WAT, and the greatest mRNA expression is found in the adipocyte [3]. This cytokine is involved in metabolic, physiological and immunological regulation in this tissue and plays a pivotal role in the production of several other cytokines (e.g., IL-10) and adipokines in WAT, such as leptin [4,5]. For instance, the anti-inflammatory interleukin 10 (IL-10) secreted by human subcutaneous and visceral adipose depots is more expressed in the latter [6]. However, tumor necrosis factor α (TNF-α), an important pro-inflammatory cytokine with a major role in the regulation of cellular processes, is secreted in a similar way by the human subcutaneous and visceral adipose depots [7]. The expression of these pro-inflammatory factors depends on the Nuclear factor kB (NF-kB) pathway. Toll like receptor 4 may activate NF-kB, which is a transcription factor and increase the pro-inflammatory response.

In stress-related situations such as exhaustive exercise and cancer cachexia, the adipose tissue shows increased expression of cytokines that act both locally and distally with autocrine, paracrine and endocrine effects [8,9]. Acute exhaustive exercise induces a pro-inflammatory response in the adipose tissue, enhancing IL-6 and TNF-α levels [8]. However, endurance exercise training induces an anti-inflammatory response in the adipose tissue, with increased IL-10 levels [10].

The effect of overload-associated exercise with a short recovery period on pro- and anti-inflammatory cytokines is however unknown. The aim of this study was to examine the effects of an overtraining protocol in the animal model upon the expression of pro- (TNF-α and IL-6) and anti-inflammatory (IL-10) adipokines, toll-like receptor 4 (TLR4) and nuclear factor kappa B (NF-kB) in depot of adipose tissue.

## Materials and methods

### Animals

Thirty-five Wistar rats, 60 d old and weighing 200-280 g were housed under environmentally controlled conditions (7:00-19:00 hr light/dark cycle; 22-24°C) and permitted free access to food and water throughout the experiment. A motorized treadmill (Columbus instruments) with 6 individual lanes without inclination was used. A shock grid at the back of the treadmill provided a mild shock (2,0 mA) if the rats pace went below the treadmill rate. The animals were familiarized with the apparatus for three days by placing them on a treadmill for 10 min/day at a speed of 12 meters/min at 0% degree inclination. To provide a measure of trainability, we rated each animal's treadmill performance on a scale of 1-5 according to the following anchors [1, refused to run; 2, below average runner (sporadic, stop and go, wrong direction); 3, average runner; 4, above average runner (consistent runner occasionally fell back on the treadmill); 5, good runner (consistently stayed at the front of the treadmill)] [11]. Animals with a mean rating of 3 or higher (n = 29) were included in the exercise groups and those with a mean rating of 1 or 2 (n = 6) were excluded from the experiment. This procedure was used to exclude possible different levels of stress between animals. All experimental protocols using animals were previously approved by the Animal Experimental Ethics Committee from Federal University of São.

### Training Protocol

The rats were subjected to 11 experimental weeks of training, as adapted from Hohl and cols [12], described in Table 1. The experimental week consisted of five consecutive days of training sessions followed by 2 days of recovery.

**Table 1:** Training Protocol

Experimental week	Training phases	**Training speed (m.min**^**-1**^**)**	Training time per session (min)	Number of daily sessions	Recovery between training sessions
1	T1	15	20	1	24
2	T1	20	30	1	24
3	T1	22.5	45	1	24
4	T1	25	60	1	24
5-8	T2	25	60	1	24
9	T2x	25	60	2	4
10	T3x	25	60	3	3
11	T4x	25	60	4	2

The physical training program was divided into 3 phases. The first phase, training phase I (T1), consisted of 4 weeks of progressively running speed and time to enhance cardiorespiratory fitness. In training phase II (T2), running intensity and duration at the end of T1 were maintained for 4 weeks to reach adaptation at a balanced running load. In the last 3 weeks of training, the frequency of daily exercise sessions was increased to two (T2x), three (T3x), and four (T4x) times adopting T2 training load. Thus, the recovery time between training sessions was reduced (4, 3, and 2 h, respectively) to induce an imbalance between overload and recovery. All training sessions were performed in the afternoon, with the first session starting at noon.

Five animals were selected for the control group (CT) and twenty- four animals for the trained (Tr) - overtrained (OT) groups and recovered overtrained protocol (R). The animals of the CT were manipulated at the same time of Tr.

### Performance Test

The training protocol (Tr) was evaluated by three performance tests. The first test was performed at the baseline after animal familiarization with the treadmill, the second test was performed at the end of T1, and the third test performed at the end of T2. The OT protocol was evaluated by three more tests at the end of the ninth 9 (T4), 10 (T5) and 11 week (T6). Thus, two additional tests were applied after the first (R1) and second (R2) recovery week. Animals were sacrificed 24 h after test T3 (n = 8Tr), T6 (n = 8Tr) and R2 (n = 8Tr n = 5CT). The testing protocol for determination the maximal effort was as follows. After a warm-up at 12 m/min on a 0% grade for 5 min, treadmill speed was increased 2 m.min-1 every 3 min until animals refused to run, which was defined as the time at which the animals could not sustain running speed without touching the shock grid ten times in 1 min. The body weight was also recorded on a digital scale before each performance test. (T0-T2, T2x-T4x, and R1-R2).

### Performance Quantification

We measured the effect of training over time. According to Hohl and cols [12] animal's performance was quantified using a mass dependent model where mechanical work is equivalent to mass × speed stage × n of minutes performed at each stage. This procedure permits the rat's performance to be measured through a quantity that is proportional to the mechanical work as shown by the respective equation

Pr=∑Pri=∑mViTi=∑mDi=mD,

where Pr represents the rat's performance; Pri is the rat's performance in each stage; m is the body mass; Vi is the stage velocity; Ti is the stage running time; Di is the stage distance; and D is the total distance covered by expressed kilogram meters (kg.m).

### Serum hormone levels

After a rest period of 24 hours after the last workout session, the animals were killed by decapitation without anesthesia. Immediately after euthanasia blood was collected and serum samples were separated after allowing blood to clot on ice. Serum was stored frozen at 80°C for analysis. Serum insulin, testosterone and corticosterone were quantified using enzyme-linked immunosorbent assay (ELISA). For insulin the kit was obtained from Millipore Corp. Bedford, MA, USA and for testosterone and corticosterone from Assay Designs, Inc., Ann Arbor, MI, USA.

### Measurement of circulating endotoxin levels

Serum endotoxin was assayed using a chromogenic limulus amebocyte lysate (LAL) test, which is a quantitative test for gram-negative bacterial endotoxin (Cambrex). Gram-negative bacterial endotoxin catalyzes the activation of a proenzyme in the LAL. The initial rate of activation is directly determined by the concentration of endotoxin. The activated enzyme catalyzes the splitting of p-nitroaniline (pNA) from the colourless substrate Ac-lle-Glu-Ala-Arg-pNA. The pNA released was measured photometrically at 405-410 nm following termination of the reaction. The correlation between the absorbance and the endotoxin concentration is linear in the 0.1-1.0 EU/ml For the purposes of this study, all samples were run in duplicate within the same plate; therefore, no interassay variability was observed in this study.

To assess recovery of endotoxin within the assay, known concentrations of recombinant endotoxin (0.25 and 1.00 EU/ml) were added to diluted, serum to determine whether the expected concentration correlated closely with the actual observed value and whether there were any variations due to reaction with serum contents. Lyophilized endotoxin (E. coli origin) was used to generate a standard curve with the chromogenic LAL test kit from Cambrex and produced a corresponding curve in accordance with the manufacturer's instructions.

### Lipid profile and liver TAG determination

Triglycerides and total cholesterol were assessed through commercial enzymatic kits (Labtest^®^, São Paulo, Brazil). Plasma glucose concentration was analysed using the enzymatic colorimetric method (Biotécnica, São Paulo, Brazil). Liver TAG content was measured with the method described by Folch et al [13].

### Measurement of TNF-α, IL-10, IL-6 protein levels

Following euthanasia, the epidydimal white adipose tissue and liver were removed, snaped frozen in liquid nitrogen, and stored at -80°C. Frozen tissues (0.1- 0.3 g) were homogenised in RIPA buffer (0.625% Nonidet P-40, 0.625% sodium deoxycholate, 6.25 mM sodium phosphate, and 1 mM ethylene-diamine tetraacetic acid at pH 7.4) containing 10 μg/ml of a protease inhibitor cocktail (Sigma-Aldrich, St. Louis, Missouri). Homogenates were centrifuged at 12.000 *g *for 10 min at 4°C, the supernatant was saved, and protein concentration was determined using the Bradford [14] assay (Bio-Rad, Hercules, California) with bovine serum albumin as a reference. Quantitative assessment of TNF-a, IL-6 and IL-10 proteins was carried out by ELISA (DuoSet ELISA, R&D Systems, Minneapolis, MN). For the TNF-α (DY510), IL-6 (DY506) and IL-10 (DY522) assays, sensitivity was found to be 5.0 pg/ml in the range of 31.2-2000 pg/ml. The intra- and inter-assay variability of the TNF-α and IL-6 kits was 2.7-5.2% and 4.9-9.5%, respectively. Assay sensitivity for IL-10 was 10 pg/ml in the range from 31.2 to 2000 pg/ml. The intra-assay variability of the IL-10 kit was 2.0-4.2%, and its inter-assay variability was 3.3-6.4%. All samples were run as duplicates, and the mean value was reported.

### Protein analysis by Western Blotting

After euthanasia, the epidydimal white adipose tissue and liver were rapidly removed and homogenized in 1.5 ml extraction buffer (100 mM Trizma, 1% SDS, 100 mM sodium pyrophosphate, 100 mM sodium fluoride, 10 mM EDTA and 10 mM sodium vanadate) and boiled for 10 min. The extracts were then centrifuged at 12,000 rpm at 4°C for 40 min to remove insoluble material. Protein determination in the supernatants was performed by the Bradford [14] dye method using the Bio-Rad reagent (Bio-Rad Laboratories, Hercules, CA, USA). The proteins were treated with Laemmli sample buffer containing dithiothreitol and boiled for 5 min before loading onto 8% SDS-PAGE in a Bio-Rad miniature slab gel apparatus. Similar sized aliquots (90 g) were subjected to SDS-PAGE as described elsewhere [15]. Electrotransfer of proteins from the gel to nitrocellulose was performed for 1 h at 120 V (constant) in a Bio-Rad miniature transfer apparatus. Nonspecific protein binding to the nitrocellulose was reduced by preincubation for 1 h at 22°C in blocking buffer (5% nonfat dry milk, 10 mM Tris, 150 mM NaCl and 0.02% Tween 20). The nitrocellulose membranes were incubated overnight at 4°C with antibodies against TLR4, NF-κBp65, Perilipin, HSL, apoB and alpha-Tubulin obtained from Santa Cruz Biotechnology (Santa Cruz, CA, USA) diluted in blocking buffer added with 1% bovine serum album (BSA)in and then washed for 30 min in blocking buffer without BSA. The blots were subsequently incubated with peroxidase- conjugated secondary antibody for 1 h and processed for enhanced chemiluminescence to visualize the immunoreactive bands. Band intensity was evaluated by optical densitometry (Scion Image-Release Beta 3b, NIH, USA) of the developed autoradiographs.

### Statistical analysis

All data are expressed as mean ± standard error (SE). Intergroup comparisons were performed by using the one-way ANOVA test. Post hoc comparison tests between groups were made using the Tukey test. The Student's *t*-test was used to evaluate the statistical significance of differences between means when need. A *P *value of less than 0.05 was considered statistically significant.

## Results

The crossover analysis of the performance of baseline, trained, overtrained and recovered groups from tests 1 to 8. We observed that performance increased progressively at test 3 and that adopting a reduced recovery time induced decreased performance in test 4. In test 5, we observed no variation of performance, and tests 6-8 showed the progressive reductions in performance characteristic of the overtraining syndrome (*data not shown*).

Table 2 shows the lipid profile, liver triglyceride content and insulin levels in all groups. Total cholesterol, glucose and insulin levels were not changed among the groups (p > 0.05), but serum triglyceride concentration was increased (27%, p < 0.05) by overtraining. Liver TAG was augmented in the overtrained (60%, p < 0.05) and recovered (80%, p < 0.05) groups when compared with the control group.

**Table 2:** Metabolic profile in all groups

Groups	TG (mg/dL)	TC (mg/dL)	Glucose (mg/dL)	Insulin (ng/mL)	Hepatic TAG (mg TAG/100 mg liver)
**Control**	136.84 ± 3.7	198.43 ± 9.5	100.50 ± 5.4	2.25 ± 0.4	10.65 ± 1.2
**Trained**	143.15 ± 7.0	172.94 ± 13.7	95.26 ± 1.7	2.36 ± 1.1	14.37 ± 2.4
**Overtrained**	174.73 ± 7.8*	166.47 ± 19.2	95.83 ± 7.3	3.01 ± 1.0	16.30 ± 2.5*
**Recovered**	148.77 ± 11.4	174.90 ± 1.5	100.75 ± 0.9	1.49 ± 0.9	18.48 ± 3.3*

Results are expressed as mean value ± SE. p < 0.05*, significantly different from control values.

**Figure 1:**
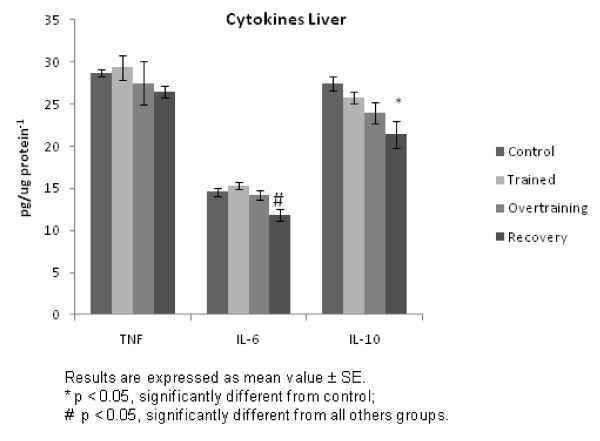
**Cytokine levels in the liver**.

TG = triglycerides; TC = total cholesterol;

Figure 1 shows cytokine expression in the liver. While TNF-α was unchanged, IL-6 was decreased (p < 0.05) in the recovered group when compared with all groups, and IL-10 protein levels were reduced (p < 0.05) in the recovered group when compared with the control.

**Figure 2:**
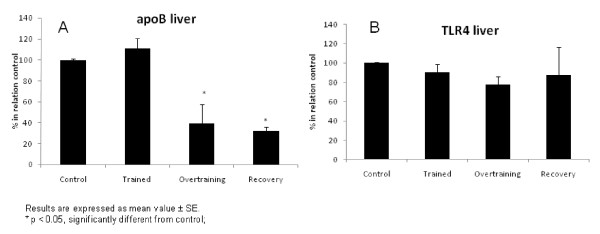
**Hepatic apoB (2a) and TLR-4 (2b) protein expression**.

Figure 2 shows hepatic apoB (Figure 2a) and TLR-4 (Figure 2b) protein expression. The overtrained and recovered groups showed diminished apoB protein expression (p < 0.05) compared with the control group. There was no change in TLR-4 among groups.

**Figure 3:**
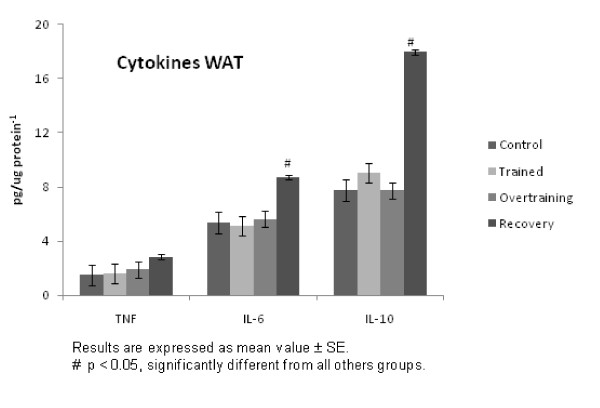
**Cytokine levels in the adipose tissue**.

Figure 3 shows cytokine levels in the adipose tissue. We found that while TNF-α was unchanged, IL-6 and IL-10 were increased (p < 0.05) in the recovered group, when compared with all other groups.

**Figure 4:**
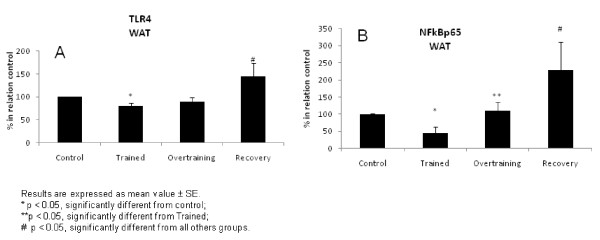
**TLR-4 (4a) and NF-κBp65 (4b) protein expression in the adipose tissue**.

Figure 4 shows TLR-4 (Figure 4a) and NF-κBp65 (Figure 4b) protein expression in the adipose tissue. TLR-4 protein expression was reduced (p < 0.05) in the trained group when compared with the control group and increased in the recovered group relative to all other groups. Reduced NF-κBp65 protein expression (p < 0.05) was found in the trained group relative to the control and overtrained groups, and the recovered group showed an increase in relation to all other groups.

**Figure 5:**
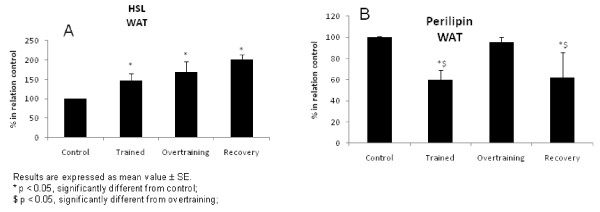
**HSL (5a) and Perilipin (5b) protein expression in the adipose tissue**.

Figure 5 shows HSL (Figure 5a) and Perilipin (Figure 5b) protein expression in the adipose tissue. HSL was increased in the trained, overtrained and recovered groups (all groups, p < 0.05), when compared with the control group. The same parameter was reduced in the trained and recovered groups (p < 0.05), when compared with the control and overtrained groups.

**Figure 6:**
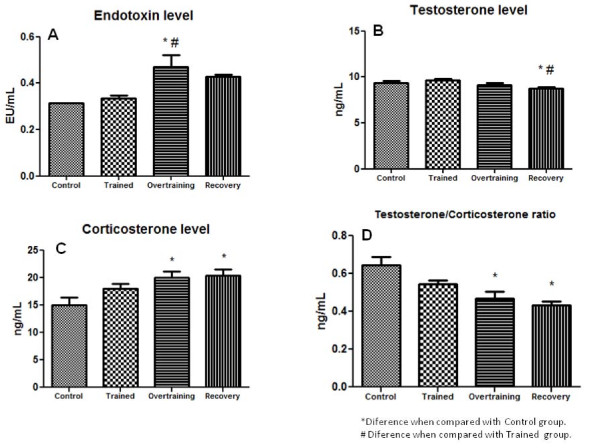
**Endotoxin and hormones levels in all groups**.

Figure 6 illustrates Endotoxin (Figure 6a), Testosterone (Figure 6b), Corticosterone (Figure 6c) levels, and Testosterone/Corticosterone ratio (Figure 6d). Endotoxin was increased in the overtrained group when compared with the control and trained groups (p < 0.05). Testosterone concentration was reduced (p < 0.05) in the recovered group when compared with the control and trained groups. Corticosterone was increased in the overtrained and recovery groups (p < 0.05) when compared with the control group. Testosterone/Corticosterone ratio was decreased (p < 0.05) in the overtrained and recovery groups when compared with the control group. (Additional files 1 and 2).

## Discussion

We have demonstrated that an overtraining protocol in rats promoted increases in both IL-6 and IL-10 protein concentration in the white adipose tissue and activated the TLR4 and NF-kBp65 pathways as well as HSL and Perilipin protein expression. The results also revealed that both IL-6 and IL-10 concentration was reduced in the liver, accompanied by lower apoB protein expression and resulting TAG accumulation (steatosis). These data were accompanied by reduced performance in exercise and lower testosterone levels, along with increased endotoxin and corticosterone levels in overtrained rats in comparison with control and trained groups.

Toll-like receptors (TLR) are trans-membrane proteins that play an important role in recognizing microbial pathogens and mediating whole body inflammation [16]. They are expressed and highly present in cells of the innate immune system [17]. TLR-4 is also found in various other cell types, including adipocytes, hepatocytes, and myocytes [18,19]. TLR-4 may activate NF-kB and increase the pro-inflammatory response. Nuclear factor kB (NF-kB) is a transcription factor associated with the expression of various pro-inflammatory factors [20]. When not stimulated, it is found in the cytoplasm connected to its inhibiting protein, inhibitor kB kinase (IkB). This complex prevents the translocation of NF-kB to the nucleus.

The endotoxemia that occurs after strenuous exercise results in increased circulating LPS and may be another mechanism underlying the elevation in TLR-4 and NF-kBp65 protein expression [21]. TLR-4 has been shown to be important in LPS-mediated responses that activate NFkBp65 via this cascade in the adipocyte [18]. Our results show that there is a increase in TLR-4 and NFkBp65 protein expression in adipose tissue after overload exercise in rats, and which might be associated with higher endotoxin levels.

In the present study, the recovered group exhibited lower testosterone and the recovered and overtrained groups presented higher corticosterone levels than the trained and control groups. The hormones control anabolic/catabolic pathways that can favour (insulin, testosterone) or antagonise (glucocorticoid hormones) [22] anabolism in skeletal muscles and with consequences to physical performance. In fact, we observed low serum testosterone and increased corticosterone levels and high testosterone/corticosterone ratios after the overtraining protocol, which could have contributed to the observed decrease in performance, and favour a pro-inflammatory status in adipose tissue.

Exercise represents a physical stress that challenges homeostasis [23]. Therefore, a single bout of exercise is a mild physical stressor that exerts an array of effects on immune parameters [24,25]. The body reacts to physical activity as it does during an acute, subclinical inflammatory response to a perceived pathological insult [26,27]. Previous studies have shown that acute exhaustive exercise induces a pro-inflammatory response in the adipose tissue, with elevated IL-6 and TNF-α levels. This increase can contribute to lipolysis and the release of fatty acids, as energy supply for muscle and other tissues immediately after exercise [8]. However, endurance exercise programs induce an anti-inflammatory response in the adipose tissue and increase IL-10 levels, leading to a protective effect [10]. Many studies [28-32] have demonstrated the benefits of aerobic training, induced by the anti-inflammatory state induced in obese rats and human models. Bradley et al [30] related that voluntary exercise in diet-induced obese mice reduced adiposity despite continued consumption of a high fat diet. In addition, exercise normalised insulin sensitivity and decreased adipose tissue inflammation (reduced IKK-β gene expression) in these animals. These results corroborate the present study, in which pro-inflammatory cytokines were reduced in the adipose tissue of the trained group.

Prolonged periods of severe training and short recovery time lead subjects to what is often called the "overtraining syndrome", which is characterized by declining performance despite an extended rest period, which is accompanied by physiological, biochemical, immunological, and psychological symptoms [33-35]. The protocol of overtraining presently adopted was able to induce the same modifications such as reduced performance found by others [12,33-35].

Several studies have explored the immune, endocrine, and psychological systems in animal and human models, with the aim of establishing key markers for the prognosis of the overtraining syndrome. The basis of Smith's hypothesis is that excessive training (high-volume/intensity training) with insufficient rest or recovery causes repetitive tissue trauma, resulting in the acute phase response and then, without adequate recovery, chronic inflammation. This state would be associated with "elevated levels of circulating cytokines (IL-1β, TNF-α, and/or IL-6), which would interact with various systems of the body and which, it will be argued, may account for most of the signs and the symptoms that previously have been associated with overtraining" [36].

There is no data in the literature about the effect of the overtraining syndrome upon cytokine production by rat adipose tissue. We show for the first time that adipose tissue contributes to cytokine production in this syndrome and that the TLR4 and NF-kBp65 pathways are involved.

In addition, we observed increased HSL in the trained, overtrained and recovered groups and decreased perilipin protein expression in the adipose tissue of the trained and recovered groups. In part, these results may explain the increase in plasma TAG, indicating exacerbation of adipose tissue lipolysis in the overtraining protocol. In fact, cytokines such as IL-6 increase lipolysis, decrease lipoprotein lipase activity and increase the mobilization of fatty acids in adipose tissue [8].

Only a small number of studies examined the effect of exercise training on perilipin expression [37,38]. Chapados et al [37] demonstrated that chronic moderate exercise (8 weeks of training) did not alter perilipin protein expression in the mesenteric adipose tissue after 8 weeks of high-fat feeding. Wohlers and Spangenburg [38] observed that perilipin protein content in mesenteric adipose tissue did not change in exercised mice.

Marked differences in gene expression among depots are reported both for rodents [39] and humans [40], and protein secretion is also heterogeneous [41]. In the present study, we demonstrated that perilipin protein expression in epidydymal adipose tissue was reduced in the trained group. However, the recovery overtrained group also showed reduced perilipin protein expression. This data may also be related to increased lipolysis and consequential contributions to increased TAG levels. Furthermore, increased plasma TAG was accompanied by reduced apoB protein expression in the liver, possibly inducing hepatic steatosis, leading the organism to chronic disease.

Taken together, our results suggest that the increase in cytokine concentration and augmented TLR-4 and NF-kBp65 protein expression induced by overtraining in the epidydymal adipose tissue may lead to a shift toward increased inflammation. Thus, recovery time from exercise training is particularly essential for the prevention of pathological conditions of chronic inflammation. The present study was the first to document the effects of an overtraining protocol on inflammatory and lipolytic status in adipose tissue in rats.

## Conflicts of interests

The authors declare that they have no competing interests.

## Authors' contributions

FSL, JCR, GDP, VAFT, RMA, FF, ESA, CON, LMO, MS, MTM and RVTS participed the sample collected, assess samples, design of the study and performed the statistical analysis, and writing of paper. All authors read and approved the final manuscript

## Supplementary Material

Additional file 1 **White Adipose Tissue**. Western blot analysis. Representative blots of three independent experiments are shown. Click here for file

Additional file 2 **Liver**. Western blot analysis. Representative blots of three independent experiments are shown. Click here for file
