# Supplementary figures and images for: Inflammation and adipose tissue: effects of progressive load training in rats
Source: Lipids Health Dis. 2010 Oct 4;9:109. doi: 10.1186/1476-511X-9-109 (PMC2959201; doi:10.1186/1476-511X-9-109)

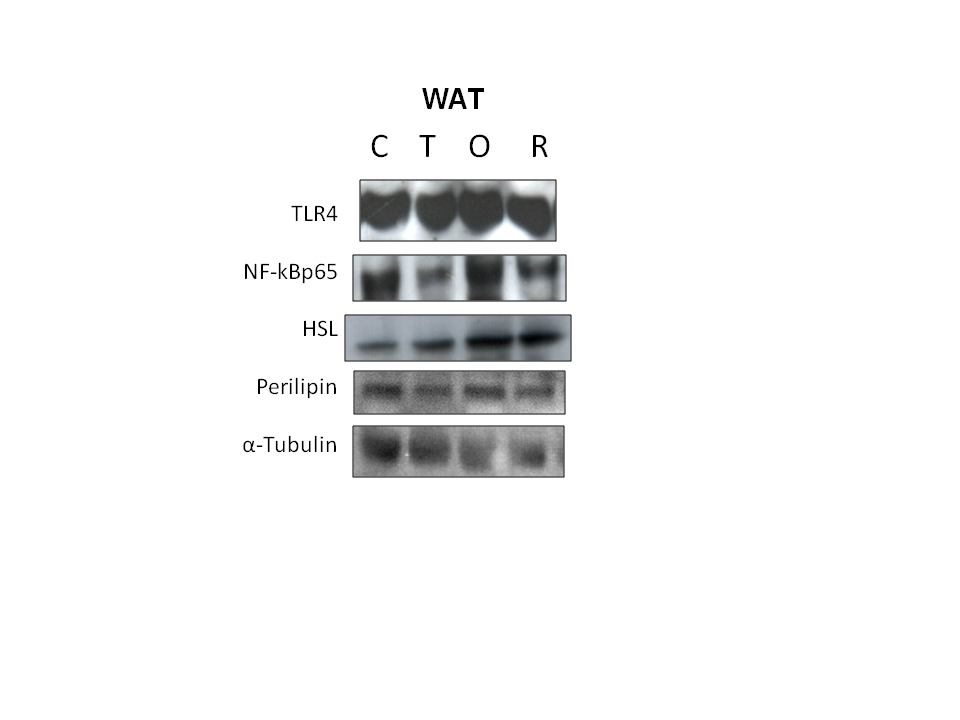

Supplement: Additional file 1 — White Adipose Tissue. Western blot analysis. Representative blots of three independent experiments are shown. [file 1476-511X-9-109-S1.TIFF]

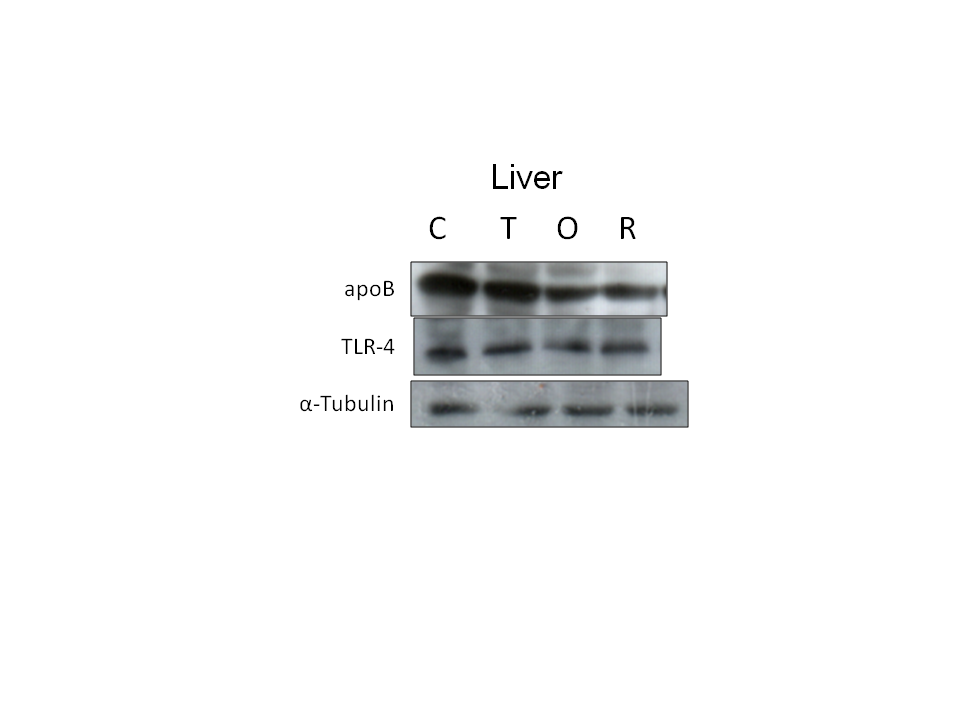

Supplement: Additional file 2 — Liver. Western blot analysis. Representative blots of three independent experiments are shown. [file 1476-511X-9-109-S2.TIFF]
